# White Pepper and Piperine Have Different Effects on Pharmacokinetics of Puerarin in Rats

**DOI:** 10.1155/2014/796890

**Published:** 2014-06-01

**Authors:** Yong-Zhuo Liang, Hai-Ming Chen, Zu-Qing Su, Shao-Zhen Hou, Xiao-Ying Chen, Yi-Feng Zheng, Yu-Cui Li, Ji Lin, Janis Ya-Xian Zhan, Zi-Ren Su, Lu-Di Fu

**Affiliations:** ^1^School of Chinese Materia Medica, Guangzhou University of Chinese Medicine, Waihuandong Road No. 232, Guangzhou Higher Education Mega Center, Guangzhou 510006, China; ^2^Laboratory Animal Center, Guangzhou University of Chinese Medicine, Waihuandong Road No. 232, Guangzhou Higher Education Mega Center, Guangzhou 510006, China

## Abstract

This study attempted to explore the effects of white pepper and its major component piperine on puerarin administered to rats. Pharmacokinetic parameters of puerarin in rats were determined by oral administration (400 mg/kg) or intravenous injection (40 mg/kg) of puerarin, pretreated with or without white pepper and piperine given orally. Compared to the control group given oral puerarin only, the combined use of piperine (10 or 20 mg/kg) increased the *C*_max_ of puerarin by 1.30-fold or 1.64-fold and the AUC_0–*∞*_ by 133% or 157%, respectively. In contrast, coadministration of white pepper (125 or 250 mg/kg) decreased oral absorption of puerarin to 83% or 74%, respectively. On the other hand, pretreatment with piperine orally did not alter the intravenous pharmacokinetics of puerarin, while the AUC of puerarin after intravenous administration was increased by pretreatment with white pepper. The results indicate that pretreatment with piperine or pepper exerts different effects on pharmacokinetics of puerarin administrated via intragastric and intravenous routes. Therefore, it is suggested that the combined application of piperine or white pepper with puerarin should be carefully monitored for potential diet-drug interactions.

## 1. Introduction

White pepper (*Piper nigrum *L.), known as “the king of spices,” is important in daily diet which helps digestion and eliminates odor and greasiness [1]. This spice has also been used as herbal medicines, preservatives, dietary supplements, and fragrances [2]. It contains components rich in aromatic oils, oleoresins, and alkaloids [3]. Piperine (1-piperoyl piperidine), an active ingredient in white pepper, has numerous biological effects such as anti-inflammatory, antioxidant, antimutagenic, and antitumor activities [2, 4]. Piperine has also been reported to promote the bioavailability of drugs such as curcumin [5], phenytoin [6], propranolol, and theophylline [7]. Its bioenhancement is due to its inhibitory activity on drug metabolizing enzymes and/or P-glycoprotein mediated drug efflux [8, 9].

Puerarin (7,4′-dihydroxyisoflavone-8-*β*-glucopyranoside) is a major active isoflavone isolated from the roots of* Pueraria lobata* (Willd.)* Ohwi.* Puerarin is commonly used for the treatment and prevention of a great variety of cardiovascular diseases in China. It has been reported to have therapeutic effects on hypertension, diabetes mellitus, arteriosclerosis, and myocardial ischemia [10–13]. However, its efficacy is therapeutically decreased due to poor water solubility and low oral bioavailability [14]. Furthermore, the short elimination half-life of puerarin in human beings causes frequent over-intravenous injection in clinics, possibly leading to various acute adverse reactions [15, 16]. There are reports of low bioavailability of puerarin that is a substrate for cytochrome P450 (CYP450) and P-glycoprotein, which are critical functional proteins in drug metabolism and transport [17, 18]. Therefore, concomitant administration of compounds that influence the CYP450 and/or P-glycoprotein would be expected to change the pharmacokinetics of puerarin.

Coadministration of plant foods with drugs is frequent, and their clinically relevant interactions are increasingly attracting researchers for the sake of public health. It was hypothesized that patients' concurrent intake of puerarin and white pepper might alter the pharmacokinetic parameters of puerarin. This might lead to an increased risk of diet-drug interactions in these patients. To date, few articles have focused on the pharmacokinetic studies of puerarin coadministrated with piperine or white pepper, and few studies have compared the effects of pepper and piperine on pharmacokinetics of a drug. Therefore, the aim of the present study was to comparatively investigate the effects of pepper and piperine on the pharmacokinetic profile of oral puerarin. Furthermore, to elucidate the function of piperine or pepper on metabolic interference, the study was extended to evaluate the intravenous pharmacokinetics of puerarin in rats after oral administration of piperine or pepper, which might offer a primary view on their pharmacokinetic interactions* in vivo.*

## 2. Materials and Methods 

### 2.1. Chemicals and Reagents

Puerarin and piperine with the purity >99% were purchased from Xian Kaicheng Biological Technology Co., Ltd. (Xian, China). p-Hydroxybenzoic acid (used as internal standard (IS)) was obtained from Shanghai Yuanye Biological Technology Co., Ltd. (Shanghai, China). White pepper obtained from Hainan Province was powdered to pass through an 80-mesh sieve. HPLC-grade methanol was purchased from Merck KGaA (Darmstadt, Germany) and acetic acid from Beijing Chemical Co. (Beijing, China). Ultrapure water was prepared using a Milli-Q gradient water purification system (Millipore, Bedford, MA, USA). All chemicals used in this study were at least of analytical grade.

### 2.2. Pharmacokinetic Study in Animals

Sprague-Dawley rats (200–220 g, half male and half female) were obtained from the Laboratory Animal Center, Guangzhou University of Chinese Medicine (Guangzhou, China). All experimental protocols involving animals and their care were approved by the Ethics Committee of Laboratory Animal Services Center. The animals were kept under controlled conditions and fed with normal standard food and tap water for 1 week. All rats were fasted overnight but supplied with water* ad libitum* prior to the pharmacokinetic investigation. To explore the effect of oral piperine and pepper on the oral pharmacokinetics of puerarin, the rats were randomly divided into five groups (*n* = 6 per group) as follows: (1) control group (pretreated with 1% tween 80); (2) low dose of piperine group (pretreated with piperine in 1% tween 80, 10 mg/kg); (3) high dose of piperine group (pretreated with piperine in 1% tween 80, 20 mg/kg,); (4) low dose of white pepper group (pretreated with pepper powder in 1% tween 80, 125 mg/kg); (5) high dose of white pepper group (pretreated with pepper powder in 1% tween 80, 250 mg/kg). Right after oral pretreatment, puerarin (400 mg/kg) was administered intragastrically to rats. Blood samples of approximately 0.25 mL were collected from the suborbital venous plexus at different time intervals (10, 20, 30, 45, 60, 90, 120, 240, 360, and 720 min) after oral administration of puerarin. To study the effect of oral piperine and pepper on the intravenous pharmacokinetics of puerarin, the rats were also randomly divided into five groups. The pretreatment groups were identical to those described above. Thirty minutes after oral pretreatment, puerarin (40 mg/kg) was administered intravenously to rats. Blood samples were obtained at predetermined intervals (2, 5, 10, 15, 25, 40, 60, 90, and 150 min) after intravenous administration of puerarin. After collection, the plasma samples were separated by centrifugation at 10,000 rpm for 10 min and stored at −20°C for subsequent analysis.

Puerarin was dissolved in propanediol for oral and intravenous administration. The dose levels of piperine were selected according to the previous pharmacokinetic studies with piperine [5, 19]. Since the content of piperine in pepper ranges from 5% to 9% [9], 125 and 250 mg/kg pepper (approximately equivalent to 10 and 20 mg/kg piperine) were employed in this investigation, which was similar to the previous study [20].

### 2.3. Preparation of Plasma Samples

An aliquot of 100 *μ*L plasma sample was added with 50 *μ*L of IS solution and 450 *μ*L of methanol, respectively. After vortex mixing for 3 min and centrifugation at 10,000 rpm for 5 min, the supernatant was separated and evaporated. The resulting dried residue was reconstituted in 200 *μ*L of methanol and then centrifuged at 2,000 rpm for 10 min. The supernatant was isolated for HPLC analysis.

### 2.4. HPLC Analysis of Puerarin

**Figure 1:**
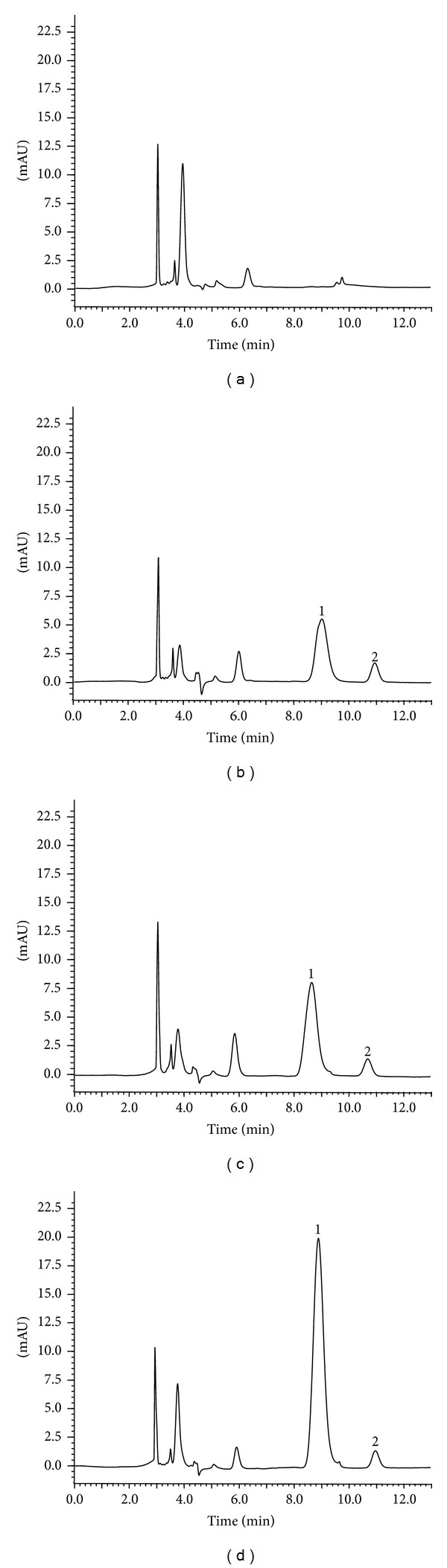
Typical chromatograms of (a) blank plasma sample; (b) blank plasma spiked with puerarin (1) and internal standard (2); (c) plasma sample from a rat at 10 min after an oral administration of 400 mg/kg puerarin; and (d) plasma sample from a rat at 10 min after an intravenous administration of 40 mg/kg puerarin.

A Shimadzu HPLC system (Kyoto, Japan) consisting of pump (LC-20AD), UV detector (SPD-20A), and LC solution chromatographic workstation was used for all analyses. The chromatographic resolution of puerarin was carried out on a Diamonsil C18 column (particle size, 5 *μ*m, 250 × 4.6 mm, Dikma, China) with the isocratic mobile phase (0.2% acetic acid in water and methanol (63 : 37, v/v)) at a flow rate of 1.0 mL/min. An aliquot of 10 *μ*L biological sample was injected for HPLC analysis, and the signals were detected at a wavelength of 249 nm. Representative HPLC chromatograms of puerarin and internal standard (IS) in rat plasma samples are shown in Figure 1. There were few interfering peaks around the retention time peaks of puerarin and the IS, with satisfactory resolution (*R* > 1.5). The calibration curves for puerarin exhibited good linearity over the concentration range from 0.8 to 500 *μ*g/mL (*r* ^2^ > 0.998).

The data for intra- and interday precision and accuracy for puerarin were measured with QC samples at three concentration levels (4,80 and 400 *μ*g/mL). The intra- and interday precision of puerarin was less than 13%, and the accuracy was between −3.8% and 9.2%. The mean extraction recoveries of puerarin at three QC concentrations were 92.02 ± 2.47%, 97.52 ± 4.13%, and 95.48 ± 3.68%, respectively. The mean recovery of IS at a single concentration was 96.93 ± 3.05%. The matrix effects on the analytes and IS indicated that the coextracted matrix produced no significant effect on the signal intensities. Puerarin at three concentrations in rat plasma was stable after three freezing and thawing cycles, at room temperature for 4 h and at −20°C for 1 week, respectively. Therefore, the proposed method was applicable to pharmacokinetic studies of puerarin.

### 2.5. Pharmacokinetic Analysis

Pharmacokinetic analysis was performed based on a noncompartmental description of the data observed. The Drug and Statistics software (version 3.1.5) was used to calculate the model-independent parameters, such as the area under the plasma concentration-time curve (AUC), the volume of distribution (*V*_*d*_), the clearance (*CL*), and the half-life (*t*_1/2_). In addition, the maximum plasma concentration (*C*_max⁡_) and the time to reach the maximum plasma concentration (*T*_max⁡_) were obtained from the plasma concentration-time data.

### 2.6. Statistical Analysis

All means were presented with their standard deviation (SD). Comparisons of the pharmacokinetic parameters were analyzed by the two-tailed unpaired Student's* t*-test, and a value of *P* < 0.05 was considered statistically significant.

## 3. Results

### 3.1. Effect of Piperine and White Pepper on Oral Pharmacokinetics of Puerarin

**Figure 2:**
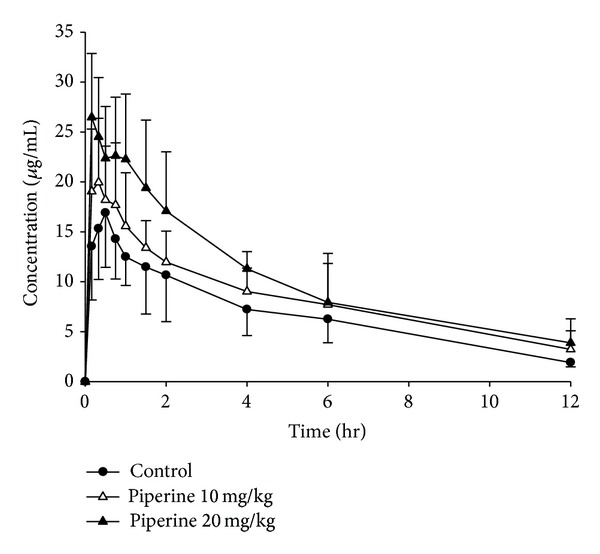
Plasma concentration-time profiles of puerarin after an oral administration of 400 mg/kg of puerarin to rats in the presence and absence of piperine. The data are expressed as mean ± SD, *n* = 6. (●) Control (combined use with tween); (△) combined use with 10 mg/kg of piperine; (▲) combined use with 20 mg/kg of piperine.

**Figure 3:**
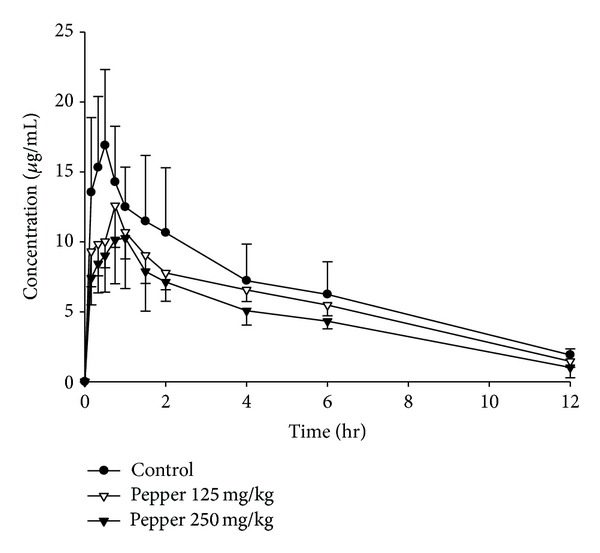
Plasma concentration-time profiles of puerarin after an oral administration of 400 mg/kg of puerarin to rats in the presence and absence of white pepper. The data are expressed as mean ± SD, *n* = 6. (●) Control (combined use with tween); (▽) combined use with 125 mg/kg of pepper; (▼) combined use with 250 mg/kg of pepper.

**Figure 4:**
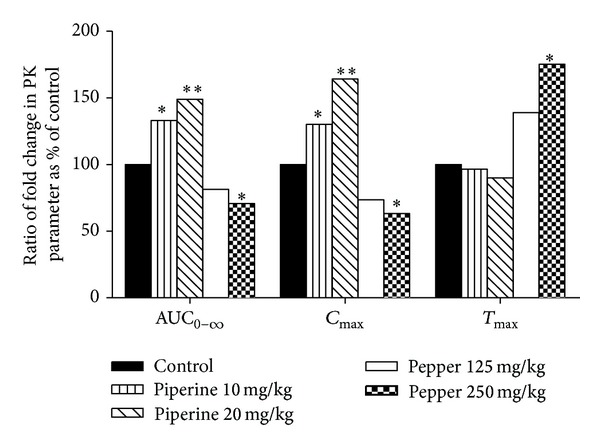
Change of the AUC_0–*∞*_, *C*_max⁡_, and *T*_max⁡_ values of puerarin as compared with control, when simultaneously administered with piperine (10 and 20 mg/kg) or pepper (125 and 250 mg/kg) and puerarin. Asterisks signs designate significant differences: _ _*P < 0.05 versus control group given puerarin only; _ _**P < 0.01 versus control group given puerarin only.

The plasma concentration-time profiles of puerarin after an oral administration at dose of 400 mg/kg, simultaneously administered with different treatments (puerarin was given following treatment), are shown in Figures 2 and 3. The pharmacokinetic parameters of puerarin are summarized in Table 1. The dose-dependent relationships of *C*_max⁡_, *T*_max⁡_, and AUC_0–*∞*_ are depicted in Figure 4. In the control group given puerarin only, the pharmacokinetic parameters of puerarin in our study was partially consistent with the result of Jiang et al. [21], in which puerarin also displayed a low *C*_max⁡_, short *t*_1/2_, and high* CL *after oral administration.

As shown in Table 1 and Figure 4, upon concomitant administration with puerarin and piperine at dosages of 10 and 20 mg/kg, the *C*_max⁡_ of puerarin was significantly increased by 130% and 164% (*P* < 0.01), respectively, as compared with control group. The AUC_0–*∞*_ of puerarin was increased by 133% or 157% (*P* < 0.01) in the presence of piperine at 10 and 20 mg/kg, respectively. In comparison to control group, *T*_max⁡_ of puerarin was decreased gradually with the increase of piperine dose, and the change of *V*_*d*_ had similar tendency. However, the difference in *T*_max⁡_, *V*_*d*_, and *t*_1/2_ between groups did not reach statistical significance, except* CL* was significantly decreased to 58% by pretreatment with 20 mg/kg of piperine.

In contrast, the *C*_max⁡_ of puerarin coadministrated with white pepper at 125 and 250 mg/kg declined to 73% and 63%, respectively, as compared to control group. *T*_max⁡_ was delayed by the combined use of pepper dose-dependently (Figure 4), which significantly increased to 0.75 h in the presence of white pepper (250 mg/kg), while the *T*_max⁡_ of control group was 0.42 h. The *T*_max⁡_ of puerarin was prolonged and the *C*_max⁡_ was decreased subjected to pepper pretreatment, indicating possible inhibition on the rate of absorbing. As listed in Table 1, coadministration with white pepper at 125 and 250 mg/kg reduced the AUC_0–*∞*_ of puerarin to 83% and 74% (*P* < 0.05), respectively. Although white pepper increased *V*_*d*_ of puerarin, it was not statistically significant (*P* > 0.05). Other pharmacokinetic parameters such as* CL *and *t*_1/2_ were not altered obviously.

Overall, these results indicated that the pharmacokinetic profiles of puerarin were changed differently when coadministered with piperine and pepper.

### 3.2. Effect of Piperine and White Pepper on Intravenous Pharmacokinetics of Puerarin

**Figure 5:**
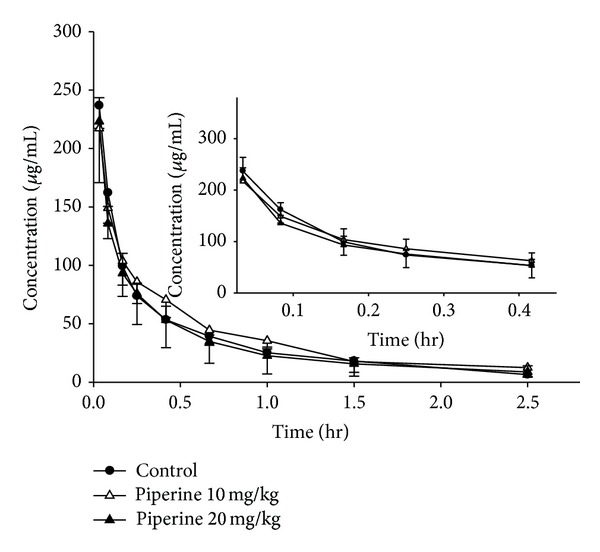
Plasma concentration-time profiles of puerarin after an intravenous administration of 40 mg/kg of puerarin to rats in the presence and absence of piperine. The data are expressed as mean ± SD, *n* = 6. (●) Control (combined use with tween); (△) combined use with 10 mg/kg of piperine; (▲) combined use with 20 mg/kg of piperine. Insert shows the pharmacokinetics of puerarin at the first 25 min after intravenous administration.

**Figure 6:**
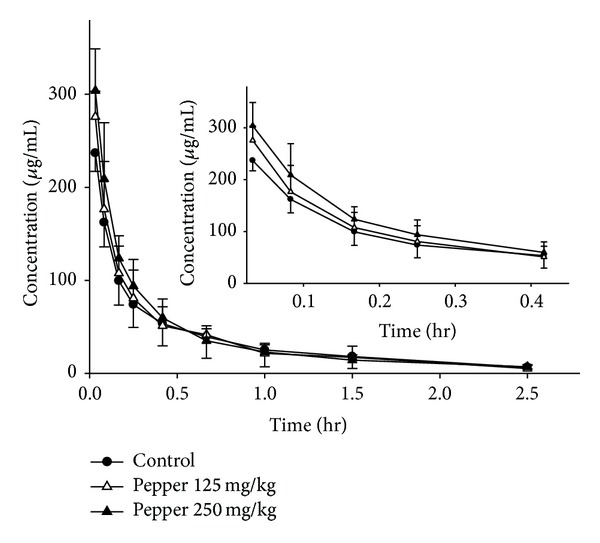
Plasma concentration-time profiles of puerarin after an intravenous administration of 40 mg/kg of puerarin to rats in the presence and absence of white pepper. The data are expressed as mean ± SD, *n* = 6. (●) Control (combined use with tween); (△) combined use with 125 mg/kg of pepper; (▲) combined use with 250 mg/kg of pepper. Insert shows the pharmacokinetics of puerarin at the first 25 min after intravenous administration.

The plasma concentration-time profiles after an intravenous administration of puerarin (40 mg/kg), with different pretreatments (30 min in advance), are shown in Figures 5 and 6. The pharmacokinetic parameters calculated from the data are summarized in Table 2. Plasma levels of puerarin declined rapidly in control group given puerarin only, which was consistent with previous result [21].

As is shown in Table 2, the* CL* and *V*_*d*_ of puerarin were not altered obviously after pretreatment with piperine. The AUC was also not significantly different in the absence and presence of oral piperine. Overall, compared with the oral pharmacokinetics of puerarin, the intravenous pharmacokinetics of puerarin was not significantly (*P* > 0.05) affected by pretreatment with piperine.

However, the combined use of white pepper (125 and 250 mg/kg) with puerarin increased the AUC_0–*∞*_ of puerarin by 115% and 128% (*P* < 0.05), respectively, as compared to the control group. Upon pretreatment with white pepper, plasma levels of puerarin at first 30 min after intravenous administration were apparently higher than those of the control group (Figure 6). The *t*_1/2_ of puerarin was increased and* CL *was reduced in the presence of white pepper, although there was no statistical significance (*P* > 0.05).

On the whole, these results indicated that the pharmacokinetic profiles of puerarin given by intravenous administration were changed by pretreatment with white pepper given orally, while it was not altered by piperine.

## 4. Discussion

Recently, the importance of natural plant products has been emphasized by the widespread recognition of diet-health linkages. White pepper, one of the most common spices, is widely used in soups, deep fried cooked foods, salads, and dressings. Although the main component of white pepper-piperine has been shown to alter the bioavailability of some drugs [5–7], very little information on the pharmacokinetic interactions of white pepper with a drug is currently available. Therefore, our study comparatively investigated the effects of white pepper and piperine on the pharmacokinetics of puerarin in rats to reveal the possible interactions.

Oral pharmacokinetics of puerarin after concomitant administration of puerarin and different treatments in rats is evaluated and summarized in Table 1. Results showed that *T*_max⁡_ was in advance, AUC and *C*_max⁡_ of puerarin was elevated in the presence of piperine. The higher plasma levels in the absorption phase and earlier *T*_max⁡_ of puerarin by the combined use of piperine might be caused by increased splanchnic blood flow and accelerated rate of transport of puerarin across the gastrointestinal mucous brought about by piperine [22]. It was reported that piperine could enhance the bioavailability of various structurally and therapeutically diverse drugs [2]. Atal et al. studied the interaction of piperine with enzymatic drug biotransforming reactions* in vitro* and* in vivo* and demonstrated that piperine was a potent inhibitor of drug metabolism [8]. Bhardwaj et al. investigated the possible effect of piperine on human CYP450 by human liver microsomes and found that piperine inhibited the major drug-metabolizing enzyme CYP3A4 [9]. It was also observed that piperine-mediated inhibition of benzo(a)pyrene metabolism was the consequence of direct interaction of piperine with the CYP1A1 enzyme [23]. From the present results, the* CL* of puerarin was significantly decreased in the presence of piperine, indicating that piperine might inhibit the elimination of puerarin. It was inferred that increased bioavailability of puerarin might be attributed to the inhibitory effect of piperine on CYP450 enzymatic activities. Since piperine did not undergo any metabolic changes in the gut [24], the luminal concentrations of piperine even at selected high dose (20 mg/kg) might not be enough for inhibition of CYP450 enzymatic activities. It is expected that the AUC of puerarin would further be increased by coadministration with higher dose of piperine.

In contrast, as shown in Table 1 and Figure 4, concurrent administration with white pepper delayed the *T*_max⁡_ and reduced the *C*_max⁡_ as well as AUC of puerarin dose-dependently. The reduction in absorption of puerarin might be explained by delayed gastric emptying and decreased gastrointestinal motility in the presence of white pepper [25]. It was reported that Trikatu, a herbal formula consisting of black pepper, long pepper, and ginger, could decrease the extent of bioavailability of rifampicin [26], which was inconsistent with results of its active principle piperine [27]. Similarly, our results showed that, although piperine was the main active ingredient of white pepper, their effects on the oral exposure of puerarin were reversed. These results might be attributable to other active components in white pepper responsible for the effect of pepper on oral puerarin. From the experimental findings (Figure 3), it was observed that the absorption of puerarin declined significantly when treated with pepper. It was hypothesized that pepper might decrease oral exposure of puerarin through inhibitory effect on intestinal absorption of puerarin, which was probably mediated by uptake transporters, as the case of phenylbutazone [28].

To further investigate the interactions of white pepper and piperine with puerarin* in vivo*, pharmacokinetic studies of puerarin after intravenous administration were performed in rats pretreated with oral white pepper and piperine. Table 2 shows that the pharmacokinetic parameters of puerarin were little affected in the presence of piperine. Considering that piperine might be metabolized within 30 min, the pharmacokinetics of intravenous administration of puerarin pretreated with piperine (5 and 15 min before) was also investigated. It turned out that the pharmacokinetics of puerarin remained unchanged in the presence of piperine. Thus, it was concluded that piperine had little effect on the intravenous pharmacokinetics of puerarin. According to the aforementioned, it was further inferred that piperine interacted with puerarin probably by inhibition of intestinal CYP450 enzymes.

On the contrary, the presence of white pepper (250 mg/kg) enhanced the AUC of puerarin significantly after intravenous administration. It was observed that excretion of puerarin was correlated to the route of administration, which was mainly excreted by intestinal tract after oral administration but dominantly eliminated by kidney after intravenous administration [29]. In addition, reports indicated that the transport of puerarin across Caco-2 cell monolayer was directional and the absorption of puerarin across intestinal sac might be an active transportation mediated by P-glycoprotein [18]. Guerra et al. showed that puerarin was a substrate for CYP450 and could obviously modulate the activity of CYP450* in vitro*, such as CYP2A1, CYP1A1/2, and CYP3A1 [17]. It was also found that puerarin acted as an enzymatic inhibitor of CYP2D6 but induced the activity of CYP1A2* in vivo *[30]. As shown in Table 2, pretreatment with pepper delayed the *t*_1/2_ and reduced the* CL* of puerarin as compared with control. These findings indicated that pepper could inhibit elimination of puerarin given intravenously, which might be due to the decrease in renal excretion or suppression of CYP450 enzymatic metabolizing activities. However, the exact mechanisms underlying this effect still remains to be elucidated.

## 5. Conclusion

The present study suggests that effects of white pepper and piperine on pharmacokinetics of puerarin in rats were significantly different. These findings indicate that the major active component might not reflect the role of the whole natural product in the pharmacokinetics of diet-drug interactions. The combined use of piperine or piperine-containing diet with puerarin should require careful monitoring for the potential interactions. As a follow-up study, we will continue to identify possible components responsible for the effect of pepper on pharmacokinetics of puerarin and to further reveal the mechanism underlying the effect of piperine and white pepper on pharmacokinetics of puerarin.

## Figures and Tables

**Table 1:** Pharmacokinetic parameters of puerarin after an oral administration of puerarin (400 mg/kg) to rats in the presence and absence of piperine and white pepper (*n* = 6, mean ± SD).

Parameters	Control	With piperine		With white pepper	
		10 mg/kg	20 mg/kg	125 mg/kg	250 mg/kg
AUC_0–*t*_ (*μ*g/mL·h)	82.059 ± 15.645	109.763 ± 21.042*	122.558 ± 25.089**	66.832 ± 12.45	58.041 ± 5.221*
AUC_0–*∞*_ (*μ*g/mL·h)	94.203 ± 17.015	125.689 ± 20.794*	148.211 ± 26.853**	78.333 ± 15.451	70.102 ± 19.051*
*C*_max⁡_ (*μ*g/mL)	18.661 ± 5.08	24.286 ± 5.662*	30.629 ± 8.636**	13.702 ± 1.557	11.789 ± 3.803*
*T*_max⁡_ (h)	0.428 ± 0.203	0.413 ± 0.272	0.385 ± 0.313	0.595 ± 0.351	0.750 ± 0.223*
*V*_*d*_ (L/kg)	27.163 ± 11.34	21.906 ± 7.086	19.675 ± 9.645	30.786 ± 6.214	33.838 ± 5.872
*CL* (L/h/kg)	4.335 ± 1.217	3.439 ± 1.158	2.525 ± 1.302*	5.087 ± 0.701	5.787 ± 1.176
*t*_1/2_ (h)	4.286 ± 0.871	4.703 ± 1.738	5.052 ± 1.797	3.678 ± 1.647	3.823 ± 0.804

Asterisks signs designate significant differences: *P < 0.05 versus control group given puerarin only; **P < 0.01 versus control group given puerarin only.

**Table 2:** Pharmacokinetic parameters of puerarin after an intravenous administration of puerarin (40 mg/kg) to rats in the presence and absence of piperine and white pepper (*n* = 6, mean ± SD).

Parameters	Control	With piperine		With white pepper	
		10 mg/kg	20 mg/kg	125 mg/kg	250 mg/kg
AUC_0–*t*_ (*μ*g/mL·h)	83.665 ± 19.132	89.287 ± 23.868	80.638 ± 15.667	95.826 ± 20.052	102.026 ± 24.22*
AUC_0–*∞*_ (*μ*g/mL·h)	90.221 ± 17.419	98.366 ± 23.219	91.982 ± 12.127	104.132 ± 21.774	115.27 ± 20.435*
*V*_*d*_ (L/kg)	0.365 ± 0.123	0.363 ± 0.07	0.402 ± 0.119	0.366 ± 0.19	0.542 ± 0.301
*CL* (L/h/kg)	0.433 ± 0.109	0.389 ± 0.118	0.441 ± 0.058	0.413 ± 0.098	0.384 ± 0.097
*t*_1/2_ (h)	0.715 ± 0.201	0.702 ± 0.204	0.652 ± 0.247	0.759 ± 0.186	0.929 ± 0.281

Asterisks signs designate significant differences: *P < 0.05 versus control group given puerarin only.
